# Standard of Spectral Radiance for the Region of 0.25 to 2.6 Microns

**DOI:** 10.6028/jres.064A.028

**Published:** 1960-08-01

**Authors:** Ralph Stair, Russell G. Johnston, E. W. Halbach

## Abstract

This paper contains information relating to the setting up of standard blackbodies for use through the temperature range of about 1,400° to 2,400° K and their use in the calibration of tungsten strip lamps as laboratory standards of spectral radiance for the wavelength region of 0.25 to 2.6 microns. A graphite blackbody is described and representative data are given on the spectral characteristics of the new lamp standard as compared to blackbodies at several selected temperatures.

## 1. Introduction

Fifty years ago the principal need in the evaluation of radiant energy was a convenient standard against which a radiometer might be calibrated. Although several crude standards of total radiance in the form of oil lamps or candles existed about 1900, the establishment of the carbon filament lamp [1, 2, 3]^1^ provided a convenient working standard for a limited range of total radiance. Then, as today, the blackbody was considered the reference standard, but its use was relegated only to the few primary scientific laboratories. The carbon filament standard has received wide acceptance in scientific research, not only in this country but throughout the world. It has been, and remains, extremely useful, but within recent years has been recognized as being inadequate to cover many new uses wherein spectral energy distributions were required.

It is to fulfill this latter requirement that a new secondary standard of spectral radiance in the form of a tungsten strip lamp has been set up. Again the blackbody has been employed as the reference source in the development of the new standard.

It has been established that the total and spectral radiation characteristics of a blackbody may be defined in terms of certain equations or laws. Planck’s radiation law relates the radiance *N*_λ_ at a particular wavelength to the absolute temperature *T* by the relationship

$$N_{\lambda }=\frac{C_{1}\lambda^{-5}}{{\;}_{e}C_{2}/\lambda T_{-1}},$$

wherein *C*_1_ and *C*_2_ are the first and second radiation constants having the dimensions watts/cm^2^ and cm/degree, respectively. The exact values attached to these depend not only on the units employed in expressing *N*_λ_, but also upon the most probable values of the various fundamental atomic constants.

In the present case the following values have been employed for the various parameters in the Planck equation:

$$\begin{array}{l} \;\lambda =\text{wavelength in cm};\\ C_{1}=1.19088\times 10^{-12}\;\text{watt}/{\text{cm}}^{2};\\ C_{2}=1.4380\;\text{cm}/{}^{\circ}K;\\ N_{\lambda }={\text{watt cm}}^{-2}\;{\text{ster}}^{-1}/\text{cm wavelength interval}. \end{array}$$

Much research has been carried out on tungsten lamps, in particular regarding the spectral emissivity of tungsten [4, 5, 6, 7, 16, 17, 18]. Although all the results are not in perfect agreement, it is generally agreed that with reasonable caution the emissivity of pure clean tungsten ribbon remains fairly constant throughout the life of the lamp. Recently, in many laboratories [8, 10, 11, 12, 15] the determination of the spectral distribution of radiant energy from a tungsten filament lamp has been obtained through making use of the published values of the emissivity of tungsten and the observed color temperature or brightness temperature of the filament. These calculations are based upon a doubtful assumption that all samples of tungsten are identical in emissivity. No account was taken of the effects of impurities present, or of the size and shape of the filament or of its mechanical or crystalline structure [4, 6]. All these properties affect markedly the true spectral and total emissivity. Furthermore, it has been found that interreflections within the lamp envelope [6] affect the total spectral radiation from a particular tungsten strip. In order to obtain the correct spectral radiance of a lamp, it becomes necessary that the particular lamp as set up for use be calibrated against a blackbody.

## 2. Apparatus and Method

Before discussing the apparatus and method employed in comparing a group of tungsten ribbon strip lamps with blackbodies at various temperatures a discussion is in order relative to the type of lamp chosen.

**Figure 1:**
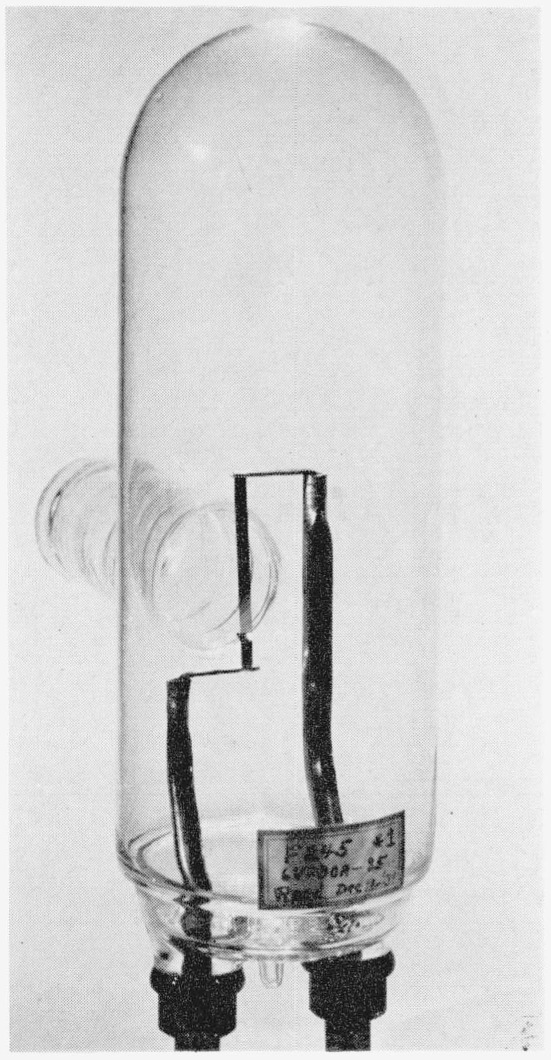
Tungsten ribbon strip lamp standard of spectral radiance.

Investigations by Worthing [4a] many years ago resulted in the accumulation of considerable information relating to the radiation characteristics of tungsten, in particular in regard to the effects of the angle (polarization, etc.) from which the filament is viewed. These results proved the necessity of using a flat-ribbon filament to insure a reproducible source. A lamp was designed at the National Bureau of Standards a number of years ago which entailed the foregoing qualities and was accepted for commercial production by the General Electric Co. as their type G.E. 30A/T24/3. This lamp was chosen for use as the new standard (see fig. 1). It has a mogul bipost base and a nominal rating of 30 amp at 6 v. Radiant energy is emitted from the flat strip filament through a 1¼-in. fused silica window placed parallel to and at a distance of about 3 to 4 in. from the plane of the filament. This separation of the window (necessitated by a graded seal) assists greatly in reducing the deposit of metallic tungsten on the lamp window as the lamp ages.

**Figure 2:**
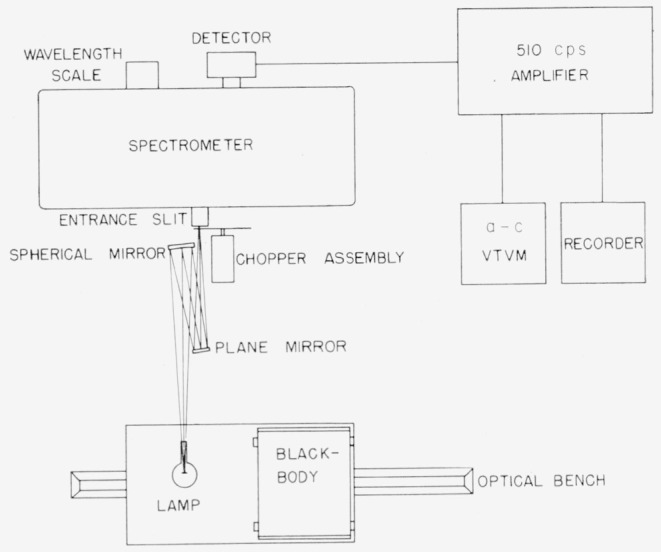
Instrumental set-up of blackbody, monochromator, lamp and associated equipment for the wavelength region of 0.7 to 2.6 microns.

**Figure 3:**
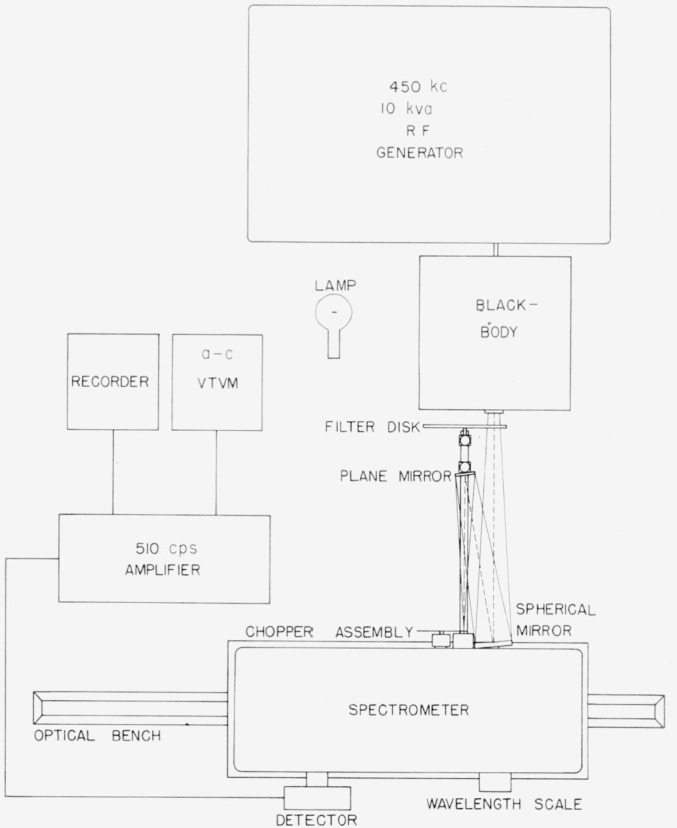
Instrumental setup of blackbody, monochromator, lamp and associated equipment for the wavelength region of 0.25 to 0.75 micron.

The principal apparatus employed in comparing the radiant energy from the lamp with that of the blackbody was set up as shown in figure 2 to cover the spectral region from 0.7 to 2.6 *μ.* The lamp and the blackbody were mounted side by side on an optical bench (constructed from a lathe bed and table) so they could alternately be placed at the focal point of the auxiliary optical system. In figure 3 is shown the monochromator mounted on the optical bench with the auxiliary optics rigidly secured on the front of the monochromator. This arrangement was employed for the spectral region of 0.25 to 0.75 *μ* wherein a high-temperature graphite blackbody was used. It was possible to move the monochromator so that the lamp and the blackbody would be alternately at the correct object distance of the auxiliary optics.

The auxiliary optics consisted of a plane mirror and a spherical mirror having a 71-cm radius of curvature. The placing of the lamp and the blackbody alternately at the same position insured equal light paths and the use of identical optics.

The blackbody indicated in figure 2, which was operated up to 1,400° K, was constructed of a casting of an alloy of 80 percent nickel and 20 percent chromium and had a 3-in. outside diameter, was 6 in. in length, and had a wall thickness of ½ in. The low reflectivity of this oxidized metal coupled with the small aperture ( 316 in. by ½ in.) as compared with the internal surface area resulted in a blackbody of extremely high effective emissivity. The high heat capacity of the associated furnace gave the blackbody a very high thermal stability, making manual temperature regulation easy. Further information on a similar blackbody employed as a standard at longer wavelengths has been published elsewhere [14].

**Figure 4:**
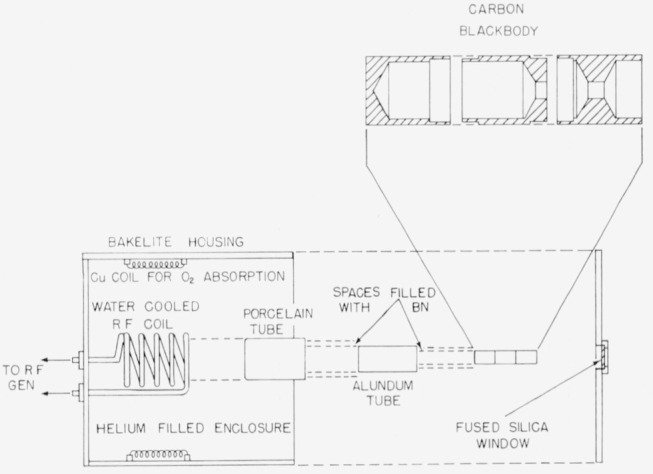
Graphite high temperature blackbody.

The blackbody employed for the shorter wavelengths and shown in figures 3 and 4 was constructed of high purity graphite. It consists of a cylindrical enclosure 4½ in. long and 1½ in. in diameter, having walls 316 in. thick. The exit end of the tube has a ⅜-in. opening shielded by a conical graphite endpiece ¾ in. long (see fig. 4.) The very low reflectivity of the graphite (rough machined surface) and the relatively small aperture (as compared to the total internal surface) resulted in a blackbody of high effective emissivity.

This graphite blackbody is heated by induction inside a water-cooled coil by a radiofrequency generator operating at 450 kc. The blackbody tube is insulated by firmly packed boron nitride powder inside a high temperature porcelain tube (closed at one end) 4 in. in outside diameter and 6 in. in length. An alundum ceramic tube placed midway between the graphite core and the high temperature porcelain tube increased the mechanical stability of the unit.

Depreciation of the graphite at high temperature was reduced by enclosing the blackbody unit in an airtight chamber (see fig. 4) through which dry helium was passed. The concentration of oxygen was further reduced by heating copper coils within the chamber preceding each operation of the blackbody.

The spectral transmittance of the fused silica window of the enclosure was measured and the necessary corrections were made for temperature measurements and spectral radiance.

This blackbody is many times larger than the small tungsten enclosures often used in high temperature work. A much larger opening can be employed thereby making possible the use of the entire slit of the usual spectroradiometer.

Leiss double quartz prism monochromators with relative aperture ranges of *F*/6.4 to *F*/7.2 were employed as shown in figures 2 and 3. The detector used at the exit slit of the monochromator for the wavelengths from 0.7 to 2.6 *μ* was an uncooled lead sulfide cell, while for the wavelength range of 0.25 to 0.75 *μ* a photomultiplier, type 1P28 was employed [9]. In all cases care was taken (through the use of filters to reduce the light intensity when required, and by other means) in using the photomultiplier to make certain that the detector was not exposed to excessive radiation which might produce a change or nonlinear character in the photoelectric response.

The radiant energy beam was mechanically chopped at 510 cps and the detector signal amplified by a special tuned amplifier [15]. Usually the output signal was read on an a-c vacuum tube voltmeter and manually recorded. However, equipment was available (and occasionally used) for recording the data with a strip chart recorder connected to the d-c output of the tuned amplifier.

The temperature of the blackbody used at 1,400° K (and below) was determined by a platinum versus platinum 10 percent rhodium thermocouple calibrated by the heat division of the National Bureau of Standards. The same laboratory calibrated the optical pyrometer employed to determine the temperatures from 1,400° to 2,400° K in the graphite blackbody. Check measurements between the optical pyrometer and the thermocouple usually were in agreement within 2° to 3° K. Calibrations of the optical pyrometer by the heat division before and following the measurements reported in this paper were in agreement to about 1° K.

Tests using thermocouples placed at various positions and by observations with the optical pyrometer indicated closely uniform temperatures within the low temperature blackbody. Similar tests with the optical pyrometer indicated a similar uniform condition within the high-temperature enclosure.

**Figure 5:**
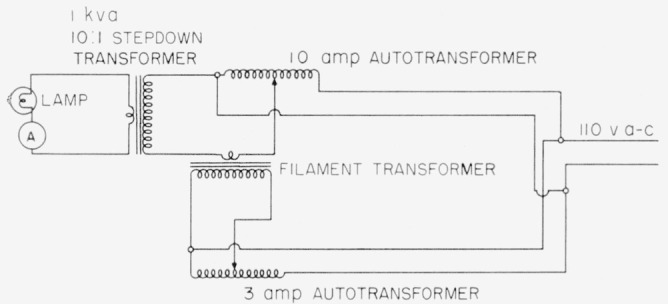
Electrical circuit for lamp operation to give smooth current control.

Each lamp was seasoned by operation on alternating current at 35 amp (about 2,470° K) for 2 hr. During calibration the lamp current was controlled manually through the use of variable and stepdown transformers as described in a later paragraph (see fig. 5).

Each lamp was set up with the envelope vertical and with the section of the filament viewed horizontally through the center of the lamp window being employed in the measurements (no note was made of a notch placed in one edge of the lamp filament for another use). Through the use of the external optics (plane mirror and spherical mirror) a full size image of this section of the lamp filament was focused upon the entrance slit of the spectroradiometer. Hence, the physical dimensions of the spectrometer slit (set at about 0.5 mm by 5 mm) determined the source area employed in the measurements. Since the optical arrangement was identical for the lamp and the blackdody equal areas of the two sources were always being compared.

Readings were alternately taken on the blackbody and on the lamp for each wavelength setting throughout the spectral range. The blackbodies were operated at several selected temperatures so that the radiance from the lamp and the blackbody were approximately equal in a given region of the spectrum. At each wavelength a number of readings were taken (with changes in the temperature of the blackbody) such that in some cases the radiance of the lamp was greater than that of the blackbody while in others it was less than that of the blackbody. This procedure enabled us to keep the effect of any departure from linearity within the electronics in the detector circuitry as small as practicable.

## 3. Results

Data obtained upon two lamps as compared with the blackbodies are given in table 1. Other lamps result in slightly higher or lower values depending upon the characteristics of the particular lamp chosen. For the laboratory lamp standards more than 20 comparisons between the lamps and blackbodies were made within the shorter wavelength region (0.25 to 0.75*μ*). About 10 to 12 independent comparisons were made at the longer wavelengths. Due to the small magnitued of scatter of the results, little is to be gained by additional measurements.

The data of table 1 are tabulated in terms of radiance, microwatts per square centimeter at a distance of 1 m from a square millimeter area source, and for a wavelength interval of 0.1*μ*. Hence the radiant energy values are in a form directly applicable for use with a spectroradiometer of fixed entrance slit. Those who desire to make solid angle measurements may change the tabulated values to erg cm^−2^ ster^−1^*μ*^−1^sec^−1^ as reported by DeVos [6] simply by multiplying the recorded value by 10^8^. Similarly, the data recorded in the DeVos tables may be reduced to the units employed herein by multiplying by 10^−8^ (within about 0.1 percent resulting from slightly different atomic constants employed in the DeVos calculations).

Although the data for all wavelengths are given on the same basis as regards the wavelength interval of 0.1 *μ*, in the experimental setup the actual instrumental spectral slit widths ranged from about 0.001 *μ* at 0.25 *μ* to 0.060 *μ* at 2.6 *μ* (being about 0.020 *μ* at 1.0 *μ*). The use of the 0.1 *μ* interval affects the absolute values hut little for wavelengths longer than about 1 *μ*. However, for the shorter wavelengths where the spectral energy curve exhibits a greater degree of curvature, higher accuracy may be obtained if the tabulated values be converted to a shorter wavelength interval corresponding more nearly to that employed in obtaining the data.

In the original comparisons with the blackbody the cone of radiant energy from the lamp was limited to approximately 5° and near normal to the plane of the lamp filament. If, in use, a larger angle is required it should be ascertained that the flux density is uniform and free of polarization throughout the added aperture.

Although the calibrations of the standards are given in terms of radiant power per cm^2^ for the various wavelengths at a distance of 1 m, the lamps may be used at any convenient distance provided proper correction is made for the new distance. However, if there is excessive water vapor in the laboratory atmosphere, errors may result at the wavelengths of water vapor absorption. In the original calibrations any water vapor absorption effects cancelled out for the greater part since the measurements were made using essentially identical air paths.

The quality of the two blackbodies used in this work was determined by the method outlined by André Gouffé [13] and the first and second order approximations of the quality of the low temperature blackbody was found to be 0.999. The quality of the high temperature graphite blackbody was 0.996.

The certification of the optical pyrometer was given as ±2° from 1,000° to 1,400° K and ±6° from 1,400° to 2,300° K. This gives rise to an uncertainty of ½ to 2 percent in the longer wavelengths while in the shorter wavelengths the uncertainty increases to 1 to 4 percent. The uncertainty for the thermocouple was less than ±3° which would result in an uncertainty in the radiance not in excess of 2 percent.

If one assumes an ability to read the meters employed as two-fifths of the smallest division, this would lead to an uncertainty never more than 1.0 percent. The ability of the electronics to reproduce an output when a given signal is applied to the input is of the order of 1.0 percent.

It is estimated that the maximum uncertainty in the results ranges from about 8 percent at the shortest wavelengths to about 3 percent at the longest wavelengths.

## 4. Use of the Standards of Spectral Radiance

**Figure 6:**
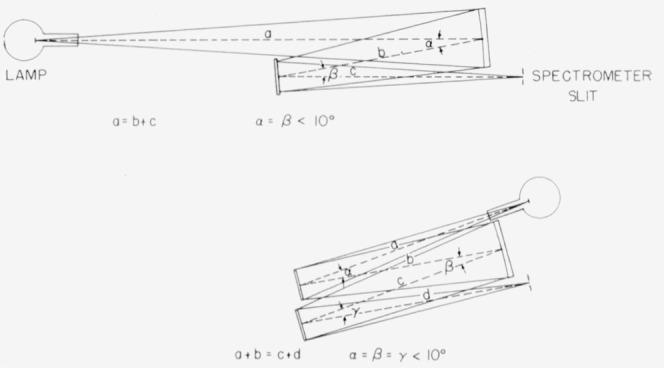
Auxiliary optics when using a lamp standard of spectral radiance in the calibration of a spectroradiometer; and for determining the spectral reflectivity of the aluminized mirrors employed.

The auxiliary optics employed with this standard may be composed of two units such as those employed in the original calibrations (see figs. 2, 3, and 6), namely a plane mirror and a spherical mirror (each aluminized on the front surface). If the spherical mirror is placed at a distance from the lamp filament equal to its radius of curvature and the plane mirror set about one-third to two-fifths this distance from the spherical mirror, and facing it (at an angle of 10° or less), an image of the filament equal in size to that of the filament itself may be focused upon the spectrometer slit. Little distortion of the filament image occurs provided good optical surfaces are employed and all reflection angles are kept to less than 10°.

In general, the radius of curvature of this auxiliary spherical mirror should be greater than the focal length of the spectrometer employed so that no loss of radiant energy will result through overfilling the spectrometer optics. Furthermore, as noted above, the aperture of this mirror should be kept within a total conical angle of about 5°.

No diaphragm or other shielding is required in the use of these standards, except for a shield to prevent direct radiation from the lamp, not falling on the concave mirror from entering the spectrometer, since in their use an optical image of the filament is focused upon the spectrometer slit.

In order to calibrate a spectroradiometer with this standard lamp, a knowledge of the spectral reflective characteristics of the mirror surfaces is required in order to evaluate the radiant energy properly at the spectrometer slit. A good aluminized surface should have a spectral reflectivity above 87 percent throughout the spectral region of 0.5 to 2.6 *μ* but which increases slightly with wavelength except for a slight dip in the region of 0.8 to 1.0*μ*. In practice the proper reflectance losses can best be determined through the use of a third mirror (a second plane mirror) which may be temporarily incorporated into the optical setup from time to time. (See fig. 6 for a possible arrangement of the auxiliary optics when including the third mirror to determine its spectral reflectance.)

In experiments where sources of radiation are being compared, no knowledge of the spectral reflectance of the auxiliary mirrors, the spectrometer transmission characteristics, or the spectral sensitivity of the detector is required. Furthermore when the same auxiliary optics are employed no measure need be taken of the spectrometer slit widths, or slit areas, provided the slit is fully and uniformly filled in both cases.

Operation of these standards should be on alternating current to obviate filament crystallizing effects which occur when the operation is on direct current. To reduce line voltage a stepdown transformer (1-kva capacity) having a ratio of 10 to 1 or a 50-amp variable transformer may be employed. (See fig. 5.) Then to give fine control a second variable transformer (10-amp capacity) is wired into the circuit to control the input of the heavy duty transformer. For still finer control a third variable transformer may be employed with a radio filament transformer to add (or subtract) a small voltage (0 to 2.5 v) to the primary voltage fed into the stepdown transformer. It was found that this method was very effective in accurately controlling the larger lamp currents. The heavy-duty (1 kva) stepdown transformer is preferred to that of a 50-amp variable transformer since the latter is subject to contact damage when operated for long intervals of time at high current values.

These lamp standards are expensive laboratory equipment and it is suggested that they be operated at the lower current value in order to prolong their usual life. Only for short intervals should they ever be operated above 30 amp (about 2,200° K), and then only to calibrate a similar lamp as a working standard. In general even at lower currents a working standard should be prepared and used, except for purposes of checking the operation of such working standard.

## 5. Conclusions

The tungsten strip lamp is a useful working standard for use in spectral radiance measurements within the region of 0.25 to 2.6*μ*. At wavelengths shorter than 0.25*μ* the available radiant energy from heated tungsten is too low for practical uses. At longer wavelengths than about 2.6*μ* the low emissivity of tungsten, together with window absorption and re-radiation effects render the use of this type of lamp impractical. Some other source, in air or employing a special window, will be found more practical. The recent measurements on the spectral emissivity of platinum from 2.0 to 15.0*μ* provides one possible standard for radiance at long wavelengths [14].

The method of calibration against a blackbody is direct and leaves no question relating to filament temperature or tungsten emissivity. The energy of the lamp is equated to that of the blackbody. The principal uncertainty in the results lie within the accurate determination of the blackbody temperatures and the measurement of the current through the lamp filament.

## Figures and Tables

**Table 1:** Spectral radiance of blackbodies and tungsten lamps= N_λ_= P×10^−q^ expressed in microwatts per square centimeter at 1 meter for a 1-mm^2^ source and for a wavelength interval of 0.1 micron

Wavelength	1,400° K blackbody		1,600° K blackbody		1,800° K blackbody		2,000° K blackbody		2,200° K blackbody		2,400° K blackbody		Lamp #20 25 amp		Lamp #20 30 amp		Lamp #16 35 amp	
*μ*	*P*	*q*	*P*	*q*	*P*	*q*	*P*	*q*	*P*	*q*	*P*	*q*	*P*	*q*	*P*	*q*	*P*	*q*
0.250	1.7493	12	2.9736	10	1.6145	8	3.9432	7	5.3871	6	4.7597	5	……	……	……	……	4.13	5
.260	6.9821	12	9.7412	10	4.5360	8	9.7969	7	1.2103	5	9.8342	5	……	……	……	……	9.59	5
.270	2.4973	11	2.9018	9	1.1720	7	2.2592	6	2.5429	5	1.9119	4	……	……	……	……	1.88	4
.280	8.1013	11	7.9434	9	2.8113	7	4.8756	6	5.0331	5	3.5211	4	……	……	……	……	3.40	4
.290	2.4084	10	2.0160	8	6.3092	7	9.9166	6	9.4458	5	6.1797	4	……	……	……	……	5.84	4
.300	6.6199	10	4.7810	8	1.3340	6	1.9128	5	1.6901	4	1.0386	3	……	……	……	……	9.72	4
.320	4.0740	9	2.2519	7	5.1031	6	6.1953	5	4.7772	4	2.6206	3	……	……	……	……	2.34	3
.350	4.0766	8	1.5976	6	2.7707	5	2.7157	4	1.7577	3	8.3334	3	……	……	……	……	7.00	3
.400	8.1943	7	2.0301	5	2.4646	4	1.8160	3	9.3067	3	3.6326	2	……	……	……	……	2.76	2
.450	7.8861	6	1.3677	4	1.2582	3	7.4259	3	3.1737	2	1.0648	1	……	……	……	……	7.27	2
.500	4.5642	5	5.9508	4	4.3847	3	2.1670	2	8.0096	2	2.3808	1	8.00	3	4.04	2	1.56	1
.550	1.8812	4	1.9255	3	1.1764	2	5.0096	2	1.6399	1	4.4073	1	1.88	2	8.14	2	2.72	1
.600	5.6287	4	4.7836	3	2.5267	2	9.5681	2	2.8441	1	7.0506	1	3.64	2	1.39	1	4.05	1
.650	1.4227	3	1.0214	2	4.7345	2	1.5838	1	4.4118	1	1.0192		6.24	2	2.13	1	5.66	1
.700	3.0045	3	1.8808	2	7.8326	2	2.4523	1	6.2392	1	1.3586		9.56	2	2.99	1	7.17	1
.750	5.6866	3	3.1435	2	1.1889	1	3.4470	1	8.2366	1	1.7026		1.35	1	3.74	1	8.59	1
.800	9.6470	3	4.8017	2	1.6731	1	4.5420	1	1.0284		2.0323		1.66	1	4.38	1	……	……
.900	2.2294	2	9.2845	2	2.8163	1	6.8433	1	1.4153		2.5938		2.22	1	5.42	1	……	……
1.000	4.1215	2	1.4883	1	4.0409	1	8.9872	1	1.7290		2.9841		2.74	1	6.12	1	……	……
1.100	6.5110	2	2.0924	1	5.1891	1	1.0736		1.9472		3.2003		3.12	1	6.38	1	……	……
1.200	9.1769	2	2.6762	1	6.1679	1	1.1992		2.0713		3.2694		3.32	1	6.41	1	……	……
1.300	1.1883	1	3.1923	1	6.8898	1	1.2761		2.1154		3.2273		3.36	1	6.24	1	……	……
1.400	1.4428	1	3.6135	1	7.3864	1	1.3103		2.0974		3.1089		3.30	1	5.94	1	……	……
1.500	1.6672	1	3.9295	1	7.6649	1	1.3101		2.0349		2.9425		3.16	1	5.55	1	……	……
1.600	1.8534	1	4.1431	1	7.7585	1	1.2839		1.9429		2.7499		2.96	1	5.04	1	……	……
1.700	1.9982	1	4.2641	1	7.7037	1	1.2393		1.8331		2.5465		2.74	1	4.52	1	……	……
1.800	2.1024	1	4.3055	1	7.5363	1	1.1825		1.7144		2.3427		2.45	1	3.94	1	……	……
1.900	2.1690	1	4.2819		7.2874	1	1.1185		1.5929		2.1454		2.20	1	3.45	1	……	……
2.000	2.2023	1	4.2071	1	6.9828	1	1.0508		1.4731		1.9585		1.97	1	3.03	1	……	……
2.100	2.2072	1	4.0941	1	6.6434	1	9.8224	1	1.3576		1.7842		1.78	1	2.65	1	……	……
2.200	2.1888	1	3.9531	1	6.2857	1	9.1466	1	1.2482		1.6235		1.58	1	2.37	1	……	……
2.300	2.1516	1	3.7930	1	5.9212	1	8.4935	1	1.1458		1.4764		1.40	1	2.13	1	……	……
2.400	2.0999	1	3.6214	1	5.5593	1	7.8705	1	1.0508		1.3425		1.26	1	1.92	1	……	……
2.500	2.0372	1	3.4432	1	5.2060	1	7.2833	1	9.6317	1	1.2211		1.13	1	1.66	1	……	……
2.600	1.9667	1	3.2633	1	4.8657	1	6.7335	1	8.8277	1	1.1113		1.02	1	1.52	1	……	……

## Footnotes

1 Figures in brackets indicate the literature references at the end of this paper.
